# Reviving Spermatogenesis: A Case Report on an Approach to Treat Non-obstructive Azoospermia Using Theophylline, Pentoxifylline, and Hyaluronic Acid

**DOI:** 10.7759/cureus.50623

**Published:** 2023-12-16

**Authors:** Charu Pareek, Ankit K Badge, Pranita A Bawaskar, Akash More, Nancy Nair

**Affiliations:** 1 Clinical Embryology, Datta Meghe Medical College, Datta Meghe Institute of Higher Education and Research (Deemed to be University), Wardha, IND; 2 Microbiology, Datta Meghe Medical College, Datta Meghe Institute of Higher Education and Research (Deemed to be University), Wardha, IND

**Keywords:** theophylline, pentoxifylline, hyaluronic acid, non-obstructive, azoospermia

## Abstract

In this report, we present the clinical management of a male patient diagnosed with non-obstructive azoospermia (NOA), a condition characterized by the absence of sperm in the ejaculate due to impaired spermatogenesis. A 37-year-old patient underwent two surgical procedures: testicular sperm aspiration (TESA) and percutaneous epididymal sperm aspiration (PESA). Surprisingly, the beta-human chorionic gonadotropins (β-HCG) testing that followed produced promising findings suggesting NOA syndrome may be reversible. Theophylline and pentoxifylline, phosphodiesterase inhibitors with immunomodulatory effects, were creatively used in this case study to increase sperm viability and activation after PESA. Hyaluronic acid was also used as an additional therapy because it is well known for aiding in sperm development and binding to oocytes. The patient underwent hyaluronic acid, which can potentially increase the fertilization rate and improve the selection of sperm. This in-depth case study offers insightful information on the effective management of NOA by combining theophylline, pentoxifylline, and hyaluronic acid. The results highlight the ability of these therapies to revive spermatogenesis, offering a cutting-edge method of treating male infertility. More research is required to clarify the underlying processes and confirm the effectiveness of this strategy in more successful reproductive medicine therapies.

## Introduction

Infertility is the inability to conceive after having frequent and unprotected sexual contact for a year or more [1]. It can be caused by several factors, such as smoking, alcohol intake, obesity, exposure to environmental pollutants, toxins, anatomical variations, impairment in drainage, and development process [2]. Ten to 15% of couples are affected by the medical condition of infertility, which sends them on an emotional journey of hope, frustration, and concern. During this challenging time, couples may seek various medical interventions, such as in vitro fertilization (IVF) or intrauterine insemination (IUI), to increase their chances of conceiving [3,4]. Comprehending the underlying obstructive and non-obstructive causes of infertility are essential to create successful treatment plans. Non-obstructive azoospermia (NOA) occurs in around 10-15% of men with azoospermia and can result from hormonal imbalance, genetic factors, and congenital anomalies [5]. Obstructive azoospermia and NOA are two primary azoospermia. Sperm production is expected in obstructive azoospermia; however, a blockage prevents sperm from getting to the semen, whereas nonobstructive azoospermia is a more complicated condition involving the testes' failure to generate sperm. Genetic predispositions, hormone imbalances, or testicular tissue injury could lead to this. Both categories provide different difficulties for naturally conceiving, but it can be overcome using assisted reproductive technologies [6]. NOA occurs when there is an impairment in spermatogenesis. This impairment can be caused by hormonal imbalances, genetic abnormalities, infections, or exposure to toxins. As a result, the production of mature and functional sperm is significantly reduced or absent [3,7].

Reproductive medicine developments, such as testicular sperm aspiration (TESA) and percutaneous epididymal sperm aspiration (PESA), have completely changed the treatment landscape and given couples new options to realize their parental goals [8]. Infertility caused by azoospermia is examined together with the astounding changes that advanced assisted reproductive technology can achieve [9]. In patients with all immotile spermatozoa, theophylline and pentoxifylline are generally used to find feasible sperm for intracytoplasmic sperm injection (ICSI) [10]. Despite being a powerful technique, the use is debatable because of the potential harm to oocytes and embryos. The mechanism of theophylline and pentoxifylline on sperm vitality involves their effects on cyclic adenosine monophosphate (cAMP) levels and inhibition of phosphodiesterase. Theophylline and pentoxifylline are known as phosphodiesterase inhibitors. These medications increase intracellular cAMP levels, improving sperm motility and vitality by promoting smooth muscle and improving blood flow. This increase in cAMP level can affect sperm movement and metabolism, which ultimately contributes to improving sperm function and viability [11].

Hyaluronic acid is a transfer medium with a significant amount of the macromolecules hyaluronan, which promotes implantation, the primary glycosaminoglycan in follicular, oviductal, and uterine fluids in hyaluronan, which largely contributes to the high viscosity environment of the female reproductive system [12]. Since then, it has been discovered that hyaluronan also encourages decidualization of the endometrial lining and that more significant amounts of the substance more accurately mimic uterine fluids and intrauterine conditions, aiding in implantation. Bicarbonate buffer medium containing hyaluronan and recombinant human albumin. It can be used for the transfer of all stages of embryo development [13].

## Case presentation

An infertile couple came to the Test Tube Baby Center of Central India for fertility treatment. Primary infertility has been the diagnosis of a 37-year-old man and 34-year-old woman for the last four years. They were both descriptively informed of all procedures, merits, and demerits, and their informed consent was obtained.

Medical History of the Couple

A 37-year-old man and his 34-year-old wife sought medical evaluation for infertility. The couple has been trying to conceive for four years with no success. They are psychologically distressed by their inability to develop and seek appropriate solutions and therapeutic choices. For the past four years, the couple has engaged in regular unprotected intercourse. Despite their efforts, they have not yet had a successful pregnancy. The female partner has a normal menstrual cycle and no documented fertility concerns, implying that the issue is most likely with the male partner's reproductive health. The male partner has no relevant medical history. He has not had severe illness, surgery, or persistent medical concerns. He denied ever having had a sexually transmitted infection (STI). No significant history of infertility or genetic disorders were reported in the immediate family. The male partner does not smoke or consume alcohol or recreational drugs. He leads a healthy lifestyle.

Clinical Findings

The male patient appeared satiated and in good general health. The vitals of the patient were within normal limits. The patient exhibited typical secondary sexual characteristics, including facial hair growth, deep voice, and average muscle mass. The genital examination revealed no abnormalities. The testicles were of moderate size and consistency with no palpable mass or tenderness. Patient semen analysis revealed the absence of spermatozoa in multiple semen samples. The patient was advised to undergo a fructose test to confirm azoospermia, and the trial resulted in a negative result after the multiple fructose test, showing the presence of fructose in the semen sample. This indicates that the patient is experiencing azoospermia, which means that there are no sperm in the ejaculate. The male patient was advised of a hormonal test, and the results showed that serum follicle-stimulating hormone (FSH) was elevated, indicating impaired spermatogenesis. The serum level of the luteinizing hormone (LH) was within the level of normal range, and the testosterone was also within the normal range (Table 1).

**Table 1:** Results of the male hormonal profile LH: luteinizing hormone, FSH: follicular-stimulating hormone, mIU/mL: milli-international units per milliliter, ng/mL: nanograms per milliliter, pg/ml: picograms per milliliter

Parameters	Reference values	Results
LH mIU/mL	0.8-7.6 mIU/mL	7.3
Testosterone ng/mL	2.50-9.50 ng/mL	7.8
Progesterone ng/mL	0.27-0.9 ng/mL	0.33
Estradiol pg/ml	20-55 pg/mL	31.73
FSH mIU/mL	1.5-12.4 mIU/mL	14
Prolactin ng/mL	<20 ng/mL	18

Treatment plan

In 2021, the patient initially went to the Wardha Test Tube Baby Centre (WTTBC). For the procedure, the couple had counseling. A quick antagonist procedure was used to begin the preparation for oocyte pickup (OPU) for the female patient. The leading follicle was stimulated with FSH/human menopausal gonadotropin (hMG) to develop to a diameter of 14-16 mm before the gonadotropin-releasing hormone (GnRH) antagonist was administered. In a short antagonist protocol, 2.5 or 5 mg/d hMG was administered in addition to human chorionic gonadotropin (hCG) to stimulate ovarian function. In conclusion, the GnRH agonist and FSH/hMG were co-administered up to the final triggers of the brief protocol. Because the GnRH antagonist causes oocyte maturation, 10,000 IU of hCG was administered 36.5 hours before ovum pick-up. Sperm extraction through PESA surgery was completed on the same day. After eight weeks of PESA, no sperm was recovered. TESA was performed and occasional and non-motile spermatozoa were recovered. An unsighted procedure called PESA was performed with oral sedation and local anesthesia. During PESA, the andrologist was able to see the exposed epididymal tubules. A 20-22 G butterfly needle was used to attach a 2 mL syringe to 1 mL of a sperm wash medium. The upper scrotum served as the entry point for the needle insertion under anesthesia. Subsequently, the syringe was employed to establish and maintain suction. A hemostat was utilized to cover the butterfly needle. The butterfly needle was then placed in the epididymis while still inside the scrotum after the tip had been placed between the forefinger and thumb. After insertion, the hemostat was removed, allowing the vacuum to push the sperm into the tubing. The needle was constantly pushed forward and backward without being removed from the skin to inject the most sperm into the tube and syringe. The sperm was placed on a glass slide by the embryologist for analysis. After pulling the incision back to reduce the chance of a hematoma, pressure was applied to the area.

Similarl to PESA, TESA also uses a tiny butterfly needle and a hemostat to maintain suction. An andrologist can handle the testes after skin blockage and sperm cord while keeping the epididymis immobile posteriorly. The scrotal skin is pulled taut before using the gun syringe to create the greatest vacuum possible. Subsequently, the needle is specifically inserted into the focal region of the middle front of the testes. The needle is kept inside the testicular parenchyma by gently moving it forward and backward while maintaining Hoover suction. An incision is removed after 10 passes while monitoring the suction. As the needle is inserted, tubules should follow. These tubules are pulled with forceps, cut, and collected on the skin's surface. Subsequently, the syringe is removed from the gun, and its suction is directed into a glass Petri dish for the embryologist to examine with a stereomicroscope. An embryologist investigated whether motile or immotile sperm was suctioned. Sperm are extracted from the surrounding tissue after being freed from the seminiferous tubules, where they had been carried. To test the viability of the sperm, theophylline and pentoxifylline are used [14].

In this investigation, theophylline solution was used and powdered pentoxifylline solution was created with a concentration of 5 mm. Five µl was used for ICSI drops, 40 µl was used for the medium in the drop that had immotile sperm in which the theophylline solution was introduced, and 10 µl was used for the medium drop where the sperm were rinsed after exposure to theophylline and pentoxifylline.

The theophylline or pentoxifylline solution was incubated at 37°C. The drop containing the sperm received 2 ml of theophylline solution and 5 ml of a 5 mm pentoxifylline solution to help activate the sperm. Theophylline or pentoxifylline was given to each 40 µl drops in cases where there were many recovered oocytes to obtain more viable sperm before looking for viable sperm for ICSI the ICSI. The plate was then set on a heated plate at 37°C for 10 minutes. The quality and morphology of the selected sperm was confirmed using a 40x lens under a microscope. Sperm that were alive and motile visibly were selected, rinsed in a medium solution immobilized in a PVP solution, and inserted into the egg. The subsequent steps, which included oocyte denudation, ICSI, embryo culture, fertilization verification, embryo development verification, and embryo transfer, followed our standard laboratory protocol.

Follow-up and outcome

**Figure 1:**
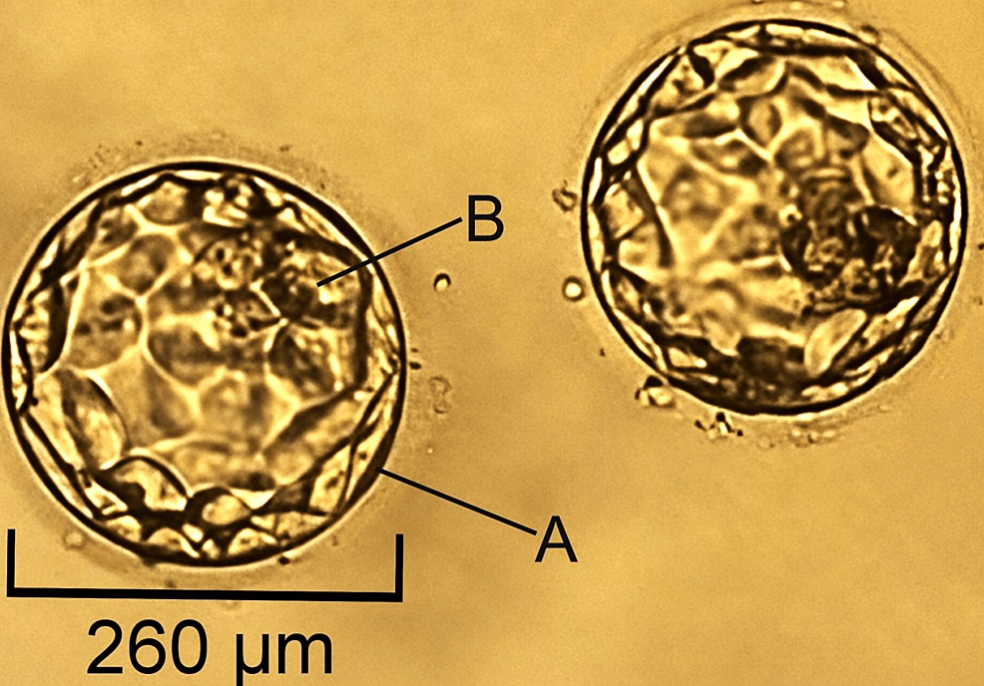
Day 5 blastocyst of grade 3 BA embryos selected for ET 3: size of the blastocyst (260 µm), B: inner cellular mass, A: trophectoderm, ET: embryo transfer

Ten oocytes were recovered in the OPU, of which eight oocytes were M2 (matured). Two oocytes were in stage M1 (immature). Eight mature oocytes underwent ICSI. Six oocytes were fertilized on day 1. Two blastocysts were formed on day 5. Cryopreserved embryos were used for embryo transfer. After two months, the embryo transfer was performed on day 12 of the menstrual cycle. Only the good-quality grade 3 BA embryos were chosen (Figure 1).

The selected embryos were placed in 50 µl of hyaluronic acid. Before transfer to the uterine cavity, the embryos were incubated for 10 minutes in the transfer medium in a 6% CO_2_ atmosphere at 37°C. The urine pregnancy test (UPT) was positive, and serum β-hCG proved a successful pregnancy with a value of 307 mlU/ml. Ultrasound sonography (USG) revealed the formation of a single sac during pregnancy. At 37 weeks of pregnancy, a healthy male baby was delivered.

## Discussion

This case study demonstrates an effective way to treat infertility caused by azoospermia, a condition in which sperm can be identified in the ejaculate. The medical intervention consisted of surgical sperm retrieval, including PESA and TESA. While in PESA, no sperm was recovered, TESA did retrieve a few nonmotile sperm [15]. Two µL theophylline and 5 µL were used to ensure the viability of the recovered sperm. Theophylline is known for its ability to improve sperm motility and increase the chances of successful fertilization. The addition of 2 µL theophylline enhanced the sperm's ability to swim and navigate through the female reproductive tract, and the 5 µL pentoxifylline solution was used to provide essential nutrients and maintain the sperm quality [16,17].

Javed et al. [18] explained that long-term exposure causes increased damage to sperm deoxyribonucleic acid (DNA), while teasing increases reactive oxygen species, all of which led to enzymatic dilution and the probability of decreased fertilization and conception, as evidenced by the increase in the live birth rate (LBR) with PESA compared to TESA. This supports our study, as TESA showed positive results compared to PESA. Mahaldashtian et al. [19] explained that the acquisition of viable sperm with pentoxifylline was more effective in terms of 2 pronuclear (2PN) and embryo development in patients with the post-thawed testicular sperm extraction (TESE) protocol without negative impacts on the integrity of sperm DNA. Oraibi et al. [20] concluded in their study that theophylline significantly reduced the time required for sperm isolation from fresh testicular samples, improved embryo quality, significantly increased implantation rate in ICSI procedures, and significantly improved biochemical and clinical pregnancy outcomes in ICSI procedures performed on male patients. Six studies [15-20] served as a basis for application in our case, as we used both theophylline and pentoxifylline to get more promising results. Thus, four embryos were developed further using both theophylline and pentoxifylline, resulting in a positive pregnancy for our patient [21].

While this case presentation presents a thorough approach to diagnosing and treating obstructive azoospermia, several limitations must be acknowledged. For instance, the effectiveness of surgical exploration and assisted reproductive procedures can vary depending on individual patient, such as the location and severity of the blockage, the quality of recovered spermatozoa, and the general health of the partner [22].

## Conclusions

The combination of surgical sperm retrieval, ICSI, cryopreserved embryo, use of hyaluronic acid for embryos, and use of theophylline contributed to the positive outcome, resulting in the birth of a healthy male baby. The case demonstrates the importance of customized fertility treatments tailored to the couple’s specific needs, offering them a chance to fulfill their need for parenthood. Furthermore, the success of this case highlights advances in reproductive medicine and the effectiveness of assisted reproductive techniques. The use of cryopreserved embryos allows better timing and planning of embryo transfer, increasing the chances of a successful pregnancy. Furthermore, the use of hyaluronic acid helps improve embryo implantation rates and improve the overall success of the IVF procedure. On the contrary, theophylline plays a crucial role in supporting embryo development and increasing the chances of a healthy birth. Technology will continue to advance in the field of reproductive medicine.
